# The impact of migrant-inclusive policy on Botswana’s universal HIV treatment program: a retrospective analysis of the national HIV treatment program data (2016–2022)

**DOI:** 10.3389/fpubh.2026.1909661

**Published:** 2026-08-24

**Authors:** Patrick T. Mokgethi, Ontlametse T. Choga, Wonderful T. Choga, Dorcas Maruapula, Natasha O. Moraka, Graceful Mulenga, Terence Mohammed, Kesaobaka Molebatsi, Catherine K. Koofhethile, Sununguko W. Mpoloka, Tafireyi Marukutira, Sikhulile Moyo, Simani Gaseitsiwe

**Affiliations:** 1 Botswana Harvard Health Partnership, Gaborone, Botswana; 2 Department of Biological Sciences, University of Botswana, Gaborone, Botswana; 3 Centre of Epidemic Response and Innovation, Faculty of Data Sciences, Stellenbosch University, Cape Town, South Africa; 4 School of Allied Health Professionals, Faculty of Health Sciences, University of Botswana, Gaborone, Botswana; 5 Department of Immunology and Infectious Diseases, Harvard T.H. Chan School of Public Health, Boston, MA, United States; 6 Directorate of Research and Innovation, University of Venda (UNIVEN), Thohoyandou, South Africa; 7 Health and Medical Research Department, Burnet Institute, Melbourne, VIC, Australia; 8 School of Health Systems and Public Health, University of Pretoria, Pretoria, South Africa; 9 Division of Medical Virology, Faculty of Medicine and Health Sciences, Stellenbosch University, Tygerberg, South Africa

**Keywords:** HIV, migrants, antiretroviral therapy, migrant inclusive policy, treat-all

## Abstract

**Background:**

Botswana has made substantial progress toward the Joint United Nations Programme on Human Immunodeficiency Virus/Acquired Immunodeficiency Syndrome (HIV/AIDS) (UNAIDS) 95-95-95 targets. However, migrants were historically excluded from free antiretroviral therapy (ART). In December 2019, Botswana extended free HIV testing and treatment to migrants. Population-level data on ART coverage, viral load testing, and virologic suppression among migrants remain limited. Therefore, the study intends to assess the impact of including migrants in the national HIV treatment program by determining ART coverage, baseline cluster of differentiation 4 (CD4) cell counts at enrollment, and virologic outcomes before and after the policy.

**Methods:**

This is a retrospective longitudinal observational dataset of all people living with Human Immunodeficiency Virus (PLWH) enrolled in the national treatment program from 2016 to 2022, with citizen status documented and aged 18 + years. The dataset was classified into two categories of individuals enrolled on ART using enrollment year: pre-2019 (citizen-only policy) and post-2019 (migrant-inclusive policy). The baseline CD4 cell count at ART initiation was compared between pre- and post-2019 eras using a modified Poisson regression and linear regression, adjusted for age, sex, and facility, with a difference-in-differences (DiD) model to provide a controlled comparison. The virologic outcomes and mortality were evaluated using modified Poisson and Cox models, adjusted for age, sex, and baseline CD4 cell counts.

**Results:**

Among 87,063 PLWH, 40,165 had baseline CD4 cell count data, while 50,043 individuals had viral load (VL) data. Median baseline CD4 cell counts among migrants were 296 cells/μL and 300 cells/μL, pre-2019 and post-2019, respectively (no significant change; *β* = 1.8 cells/ mm^3^, 95% confidence interval [CI]: −14.0 to 17.6; *p* = 0.819). Advanced HIV disease at ART initiation (CD4 < 200 cells/μL) declined significantly from 22.9 to 17.7% (adjusted relative risk [aRR]: 0.76, 95% CI: 0.63–0.90; *p* = 0.002). Difference-in-differences models showed more favorable changes post-2019 among migrants vs. citizens in both continuous CD4 cell counts (interaction *β* = 54.2 cells/mm^3^, 95% CI: 37.7–70.6; *p* < 0.001) and advanced disease at initiation (interaction RR = 0.74, 95% CI: 0.62–0.89; *p* = 0.001). The VL measurement capture among migrants increased from 27.7 to 58.5% at 6 months, 31.1–66.1% at 12 months, 32.0–74.6% at 24 months, and 40.5–82.6% at 36 months (all adjusted RRs: 1.86–2.25; *p* < 0.001) after the policy shift. Observed virologic suppression (<400 copies/mL) remained high (≥94%) with no significant post-2019 change. Mortality was low and unchanged post-2019 (interaction adjusted hazard ratio [aHR] 1.99, 95% CI: 0.41–9.70; *p* = 0.393).

**Conclusion:**

Extending Botswana’s ART program to migrants reduced advanced HIV disease at initiation and significantly improved viral load monitoring and sustained viral suppression. Removing citizenship-based barriers reduces health inequities and supports epidemic control toward the UNAIDS 95-95-95 targets.

## 1 Introduction

Botswana, a country with one of the highest prevalence rates (20.8%) of Human Immunodeficiency Virus (HIV), has made remarkable efforts in response to the HIV epidemic with a robust HIV treatment program (1, 2). Botswana was among the first countries to offer free antiretroviral therapy (ART) to its citizens (3) since 2002 (3).

Over the years, Botswana continued to make strong strides in the fight against HIV. In 2016, the government adopted the World Health Organization’s “Treat-all” HIV treatment recommendation guidelines to provide treatment to all people living with Human Immunodeficiency Virus (PLWH) regardless of their HIV viral load levels or cluster of differentiation 4 (CD4) cell counts (4). Additionally, the adoption of dolutegravir-based ART as part of the first-line treatment regimen was instrumental toward achieving the Joint United Nations Programme on Human Immunodeficiency Virus/Acquired Immunodeficiency Syndrome (HIV/AIDS) (UNAIDS) ambitious 95-95-95 targets (4–6). However, the Botswana “Treat-all” policy program excluded migrants living with Human Immunodeficiency Virus (MLWH) (except for refugees in camps and prisoners) (7, 8). Migrants constitute a substantial proportion of the population of Botswana (7–9). Botswana reported 110,268 migrants, with an estimated 30,000 (~27%) living with HIV (10, 11). Migrant Populations remain marginalized and frequently face poor health outcomes due to restrictive laws and policies, limited access to health care services, vulnerability to exploitation, stigma, and discrimination (9, 12, 13). Due to the aforementioned factors, migrant populations remain vulnerable to poorer health outcomes and face higher HIV transmissions, which undermines public health goals (14–16).

The association between health and migration is important, given that Botswana is a landlocked country with high HIV prevalence and surrounded by countries with also high HIV prevalence (13, 16–18). In December 2019, the government of Botswana took an important step and extended its national free ART program to include all MLWH (8, 16). Taking this decision was a major step by the government toward universal health coverage and recognizing the human rights implications and public health risks of excluding migrants from HIV treatment and care services, in efforts to achieve UNAIDS targets (1, 13, 16, 19). Currently, there is limited evidence on the impact of the migrant-inclusive Treat-All policy on the HIV treatment and care outcomes among migrants in Botswana. It is essential to measure the outcomes of the migrant-inclusive policy and determine how the country’s UNAIDS 95-95-95 targets have been affected and how they can be improved. Therefore, the national treatment program data provide an opportunity to assess the impact of including migrants in the Botswana national treatment program by evaluating the clinical outcomes of migrants compared to citizens.

We conducted a retrospective longitudinal observational analysis of national HIV surveillance data to evaluate HIV care among migrants compared to citizens before and after the December 2019 policy change, and to quantify changes in ART coverage and clinical and virologic outcomes following policy implementation.

## 2 Methods

**Figure 1:**
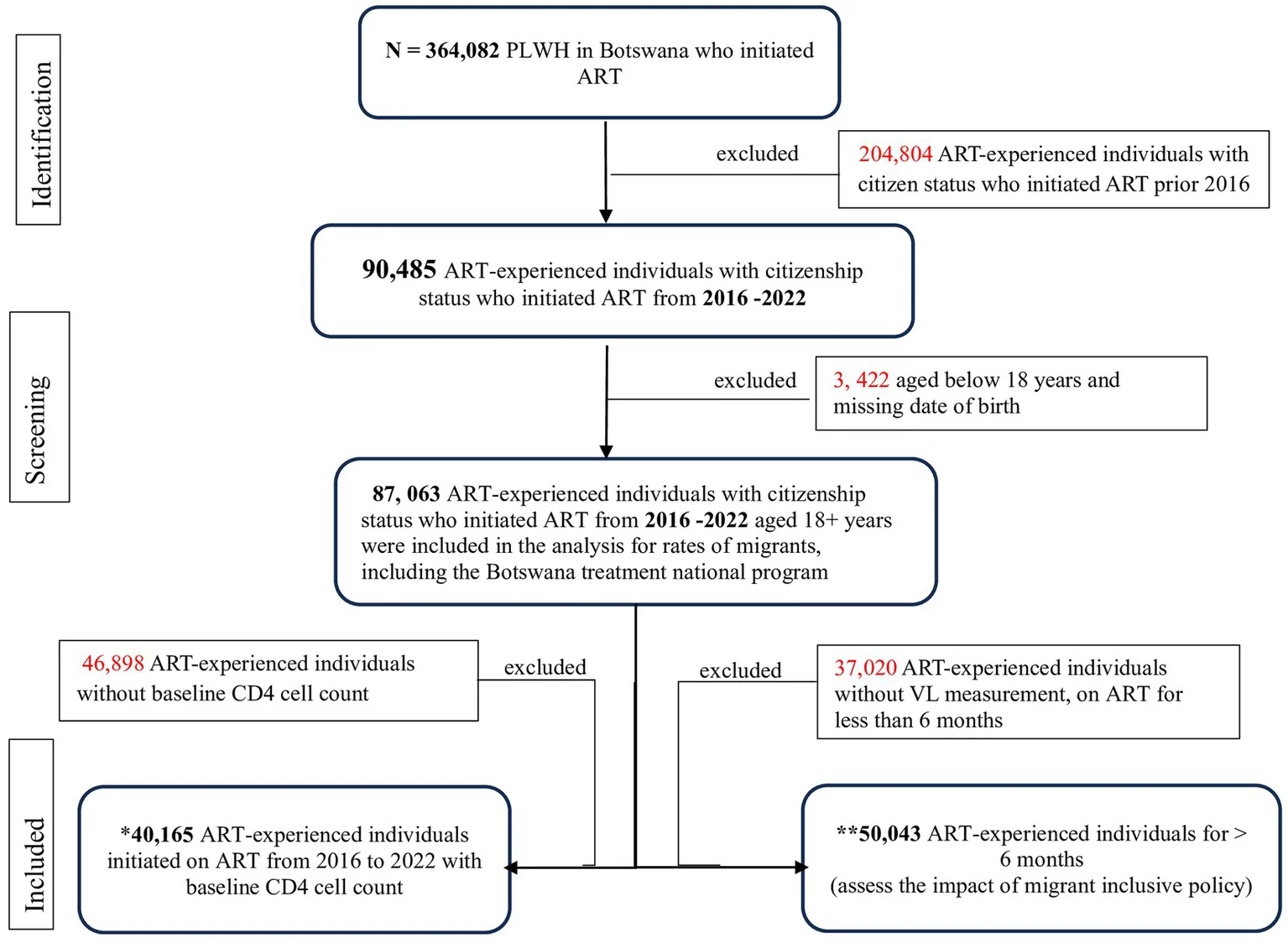
Study inclusion criteria analysis flow diagram. *Participants included in the baseline CD4 cell-count analysis (*n* = 40,165); **Participants included in the viral-load analysis after being on ART for >6 months (*n* = 50,043).

Study design and population: This is a retrospective longitudinal observational study utilizing all people living with Human Immunodeficiency Virus (PLWH) enrolled in the Botswana national treatment program from 2016 to 2022, aged 18 + years, with documented citizenship. Clinical information from all PLWH in Botswana was captured in the electronic medical records, which are used in routine clinical care and operational monitoring and assessment of the HIV treatment program. The data were classified into two categories of individuals enrolled on antiretroviral therapy (ART) using enrollment year: pre-2019 (2016–2019) (ART free for citizen-only policy) and post-2019 (2020–2022) (ART free for all, inclusive of migrants). Selection of study participants: Analysis was restricted to individuals with documented citizenship status, enrolled in the national program from 2016 to 2022, and aged 18 years or older. The other inclusion criteria were having a baseline CD4 cell count, which was utilized in the analysis specific to it. Additionally, PLWH on ART for at least 6 months with at least one viral load (VL) measurement were considered for this analysis (Figure 1). Of 87,063 adults included overall, 40,165 contributed to baseline CD4 analyses, and 50,043 contributed to viral load analyses.

### 2.1 Data quality assurance

In line with the standard operating procedures at the National Data Warehouse, the workflow for deduplicating data across systems such as Integrated Patient Management Systems (IPMS), Patient Information Management System (PIMS), and electronic medical records (OpenMRS) is conducted to maintain high data quality and structural integrity across millions of health records. PIMS backups are performed monthly and securely transferred to the National Data Warehouse, where they are combined with other data. Processes such as data cleaning and completeness checks are implemented to improve data quality.

### 2.2 Definition of study exposures and outcomes

The primary study exposures were citizenship status and the time of ART initiation. Citizenship status was based on the routinely recorded national database citizenship field and categorized as citizen vs. non-citizen (migrant). The ART period was categorized into two policy eras based on the timing of Botswana’s HIV treatment policy change: pre-inclusion policy period (2016–2019), during which ART coverage was restricted to citizens, and post-inclusion policy era (2020–2022) where ART treatment access was expanded to migrants. The primary study outcomes were advanced HIV disease at ART initiation, viral load (VL) capture, and viral suppression. Advanced HIV disease stage was defined as a baseline CD4 cell count below 200 cells/mm^3^. VL capture referred to the availability of at least one VL measurement at 6 months on ART. Viral suppression was defined as VL ≤ 400 copies/mL after 6 months on ART, while virologic failure was VL > 400 copies/mL. Individuals without a VL in the window remained missing and were not classified as unsuppressed in the primary analysis.

CD4 cell counts and viral load were measured in different laboratories in Botswana using several assays with different lower limits of detection. The Botswana HIV guidelines recommend CD4 cell count testing at baseline, at 6 months post-ART initiation, and if the CD4 cell count is <200 cells/mm^3^, then annually. HIV VL is measured at 3 months post-ART, then every 6 months after viral suppression (20). Individuals who are not suppressed on VL measurement at 6 months are considered virologically failing after a confirmatory VL is completed. VL testing will then be repeated at 4–6 weeks, and those who have 1Log10 drop will continue with frequent VL measurements at 2-week intervals until viral suppression is achieved. Botswana’s national HIV clinical guidelines recommend directly observed therapy (DOT) for individuals who experience confirmed virologic failure. VL testing will be performed after 30 days on DOT, and if suppression is not achieved, HIV drug resistance testing will be conducted. PLWH who are virally suppressed will then have their VLs assessed at 6-month intervals. The secondary outcome was defined as mortality among PLWH with citizenship status, aged 18 + years, and with at least one VL measurement.

### 2.3 Statistical analysis

Baseline demographics were summarized using descriptive statistics by the year of ART initiation, citizenship status, and policy era. Continuous variables were reported as medians with first and third quartile ranges, while categorical variables were reported as percentages. Among PLWH with baseline CD4 cell counts, changes in baseline CD4 cell count before and after policy inclusion were evaluated by comparing changes in continuous CD4 cell counts using a linear regression model. Advanced HIV disease at ART initiation (CD4 < 200 cells/mm^3^) was analyzed using a modified Poisson regression with a log link to evaluate differences in HIV disease stage across policy periods and citizenship groups.

A difference-in-differences (DiD) model was used to provide a controlled comparison between citizenship and policy eras and to assess whether observed changes in migrants differed from concurrent changes in citizens. The inclusion of migrants in the national treatment program in 2019 was defined as the policy intervention, which was classified into the pre-policy period (2016–2019) and the post-policy period (2020–2022). The DiD method assessed whether the policy changes significantly affected outcomes among migrants and whether the clinical and virologic outcomes were similar in migrants and citizens after the policy change. To assess the plausibility of the difference-in-differences assumptions, we conducted supplementary pre-policy diagnostics for baseline CD4 outcomes. First, we graphed annual pre-policy trends (2016–2019) for continuous baseline CD4 at ART initiation and for advanced HIV disease at ART initiation (CD4 < 200 cells/mm^3^), stratified by citizenship. Second, restricted to pre-policy years only, we fitted interaction models including citizenship, calendar year, and citizenship × calendar year terms, adjusted for age and sex. For continuous baseline CD4, we used linear regression with robust standard errors; for advanced HIV disease at ART initiation, we used modified Poisson regression with robust standard errors. The year 2016 was used as the reference category, and we tested whether the pre-policy citizenship × year interaction terms were jointly equal to zero.

The rates of VL capture and VL suppression were determined at prior and post-policy inclusion, focusing on PLWH with VL measurement after 6 months on ART with at least one VL measurement. These rates were further assessed by ART initiation period (2017–2022) and at 6-month intervals (6, 12, 24, and 36 months post-ART initiation). The participant-specific window records at 6, 12, 24, and 36 months after ART initiation were created, and the VL closest to each target month within a prespecified ±3-month window was selected. When multiple VL measurements were available in the timepoint strata, the latest VL measurement was utilized. The adjusted modified Poisson models were fitted to compare post-2019 vs. pre-2019 migrants for both VL capture and observed suppression. The adjusted variables included citizenship, a citizenship × post-2019 interaction, adjusted for age, sex, and baseline CD4 cell counts. Mortality rates were summarized as crude rates per 100 person-years and stratified by citizen status and policy eras. Predictive factors for all-cause mortality were analyzed using the Cox proportional hazards regression model. All models were adjusted for citizen status, age, gender, and baseline CD4 cell count. In the analysis, PLWH who were alive were right-censored after their last available VL test. The results were reported as hazard ratios indicating the risk of all-cause mortality for each variable compared to their reference group. All tests were two-sided, and confidence intervals were reported at the 95% level. The proportional hazards assumption was checked graphically and using Schoenfeld residuals tests. All statistical analyses were performed using STATA version 19, and a *p*-value≤0.05 was considered statistically significant.

## 3 Results

### 3.1 Overall demographic characteristics and rates of yearly ART initiation by citizenship in the Botswana national treatment program (2016–2022)

A total of 87,063 PLWH met the study inclusion criteria (Figure 1). Among 87,063 included individuals, the overall prevalence of migrants enrolled in the national program was 5,573 (6%) (Table 1). Of 87,063 adults included overall, 40,165 contributed to baseline CD4 analyses, and 50,043 contributed to viral load analyses. Overall, the number of PLWH initiating ART declined sharply over the period, from 22,892 in 2016 to 5,682 in 2022. Migrants who initiated ART were few in the early years (1.3–4.3% in 2016–2019), but increased substantially from 2020 onwards, reaching around 19–20% in 2020–2022. Females consistently accounted for the majority of PLWH initiating ART (~57–65%), though this proportion declined slightly over time. Median age at ART initiation remained stable throughout, at 32–33 years (IQR: ~25–41), and the majority were unmarried PLWH in all ART initiation years.

**Table 1:** Demographic characteristics and yearly ART initiation rates (2016–2022).

Characteristic	Year of ART initiation							Total *N* (%)
	2016 *n* (%)	2017 *n* (%)	2018 *n* (%)	2019 *n* (%)	2020 *n* (%)	2021 *n* (%)	2022 *n* (%)	
Total initiations	21,903	18,929	14,385	10,933	8,383	7,013	5,517	87,063
Sex								
Female	14,238 (65.0)	11,378 (60.1)	8,202 (57.0)	6,283 (57.5)	4,889 (58.3)	3,997 (57.0)	3,135 (56.8)	52,122 (59.9)
Male	7,665 (35.0)	7,551 (39.9)	6,183 (43.0)	4,650 (42.5)	3,494 (41.7)	3,016 (43.0)	2,382 (43.2)	34,941 (40.1)
Citizenship								
Citizen	21,606 (98.6)	18,637 (98.5)	14,109 (98.1)	10,451 (95.6)	6,669 (79.6)	5,590 (79.7)	4,428 (80.3)	81,490 (93.6)
Migrant	297 (1.4)	292 (1.5)	276 (1.9)	482 (4.4)	1,714 (20.4)	1,423 (20.3)	1,089 (19.7)	5,573 (6.4)
Age at diagnosis (years)								
Median (IQR)	33 (26–41)	33 (26–41)	33 (26–41)	33 (26–41)	34 (27–41)	34 (27–41)	34 (27–42)	33 (26, 41)
Marital status								
Single	16,376 (91.2)	14,120 (92.2)	10,763 (93.2)	8,027 (92.4)	5,854 (90.9)	4,860 (90.2)	3,773 (91.9)	63,773 (91.8)
Married	1,436 (8.0)	1,083 (7.1)	724 (6.3)	615 (7.1)	560 (8.7)	502 (9.3)	309 (7.5)	5,229 (7.6)
Divorced/separated/widowed	146 (0.8)	112 (0.7)	59 (0.5)	43 (0.5)	29 (0.4)	29 (0.5)	23 (0.6)	441 (0.6)

Marital status data available for 69,443 of 87,063 individuals (79.8%); IQR, interquartile range; ART, antiretroviral therapy. The decline in annual ART initiations over time likely reflects maturation of the national Treat-All programme, with fewer untreated individuals remaining to initiate ART in later years, as well as evolving program coverage and testing patterns.

### 3.2 Geographic distribution proportions of participants by citizenship

**Figure 2:**
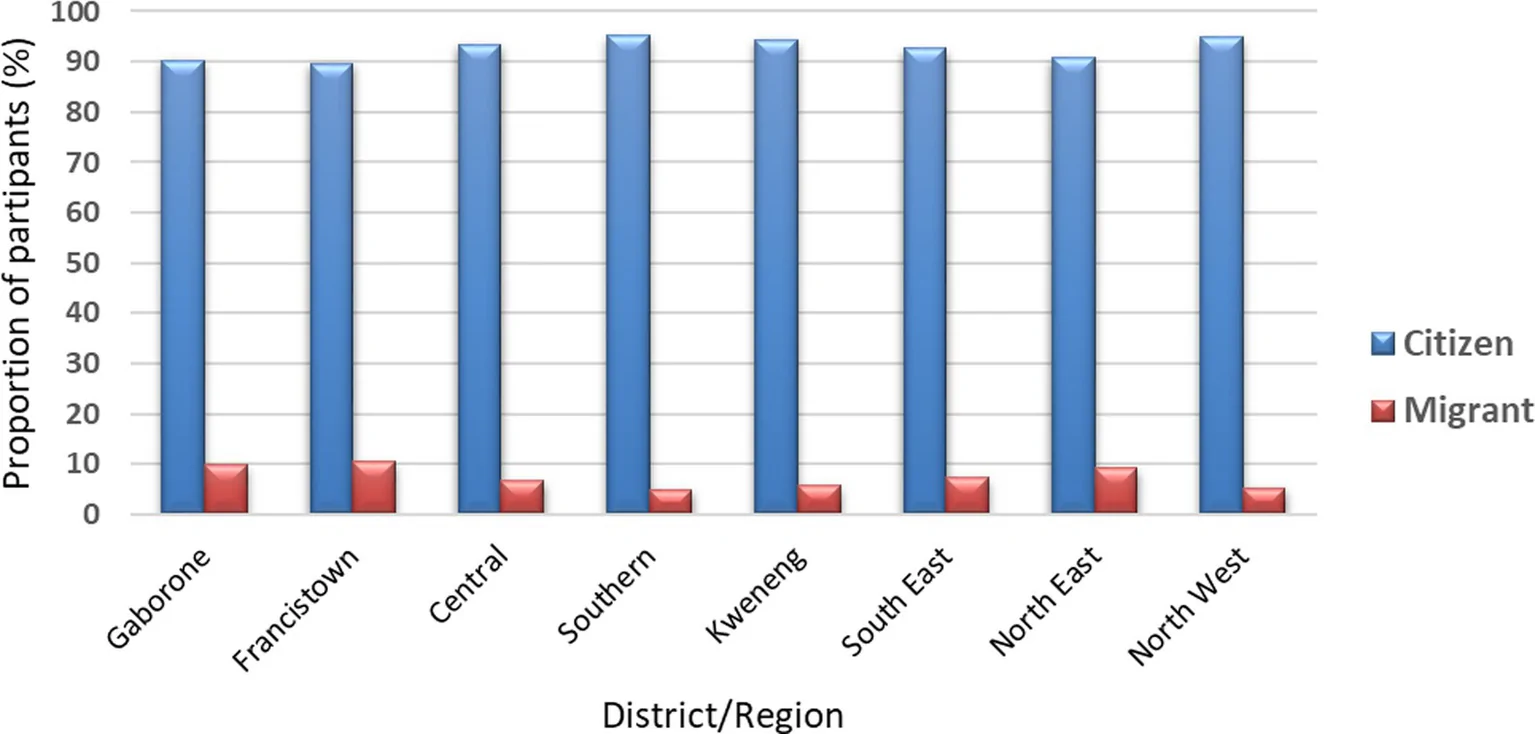
Geographic distribution of participants by citizenship. Health facilities were grouped according to their respective administrative districts. Gaborone and Francistown accounted for the largest participant populations. Regions were classified as *urban* (Gaborone and Francistown), *Peri-Urban* (Central and South East), or rural (Southern, Kweneng, North East, and North West) based on Botswana’s administrative classification, where cities are considered urban, and districts are considered rural.

Figure 2 illustrates the regional distribution of study participants by citizenship. The migrants represented a larger proportion of participants in urban areas like Gaborone and Francistown. Migrants were also prevalent in regions closer to the borders, such as in the North-East region.

### 3.3 Comparison of baseline CD4 cell counts at ART initiation pre-2019 and post-2019

A total of 40,165 ART initiators (1,994 (5%) migrants and 38,171 (95%) citizens) had a baseline CD4 cell count. Among migrants, the median baseline CD4 at ART initiation increased from 296 cells/μL before 2019 to 300 cells/μL after 2019. The change in median CD4 cell count post-2019 was 1.8 cells/μL (95% CI: −14.0 to 17.6; *p* = 0.819). The proportion of migrants initiating ART with CD4 < 200 cells/μL declined from 22.9 to 17.7%, and the relative risk of initiating ART among migrants presenting advanced HIV disease post-2019 was 0.76 (95% CI: 0.63–0.90; *p* = 0.002). Table 2 shows the characteristics and CD4 cell counts of individuals included in the analysis by citizenship and policy era.

**Table 2:** Baseline characteristics of people living with HIV initiating ART by citizenship and policy era.

Characteristic	Migrants (non-citizens)			Citizens		
	Pre-2019 (*n* = 573)	Post-2019 (*n* = 1,421)	*p*-value^a,d^	Pre-2019 (*n* = 28,854)	Post-2019 (*n* = 9,317)	*p*-value^a^
Age at initiation, median (IQR)	36 (31, 42)	37 (31, 43)	0.036	33 (26, 41)	33 (26, 41)	0.009
Female, *n* (%)	285 (49.7%)	690 (48.6%)	0.633	17,829 (61.8%)	5,437 (58.4%)	<0.001
Baseline CD4, median (IQR)	296 (200, 400)	300 (200, 364)	0.752	336 (200, 513)	292 (200, 422)	<0.001
CD4 < 200 cells/μL, *n* (%)	131 (22.9%)	252 (17.7%)	0.009	6,866 (23.8%)	2,329 (25.0%)	0.018
ART regimen at ART initiation, *n* (%)^b,c^						
DTG-based	377 (65.8%)	1,170 (82.3%)	<0.001	18,324 (63.5%)	7,147 (76.7%)	<0.001
EFV-based	130 (22.7%)	112 (7.9%)	<0.001	5,278 (18.3%)	776 (8.3%)	<0.001
NVP-based	3 (0.5%)	3 (0.2%)	0.363	328 (1.1%)	47 (0.5%)	<0.001
PI/boosted-based	5 (0.9%)	1 (0.1%)	0.009	184 (0.6%)	16 (0.2%)	<0.001
Missing	56 (9.8%)	132 (9.3%)	0.738	4,651 (16.1%)	1,311 (14.1%)	<0.001

ᵃ*p*-values compare Pre-2019 vs. Post-2019 within each population separately (migrants and citizens); they do not compare migrants with citizens. Kruskal–Wallis test for continuous variables (age, baseline CD4); Pearson chi-square for categorical variables, with Fisher’s exact test where any expected cell count was <5 (NVP- and PI/boosted-based rows among migrants). Computed on participant-level data (*n* = 40,165). ᵇRegimen categories do not sum to the column totals: a further 2 (0.3%), 3 (0.2%), 89 (0.3%) and 20 (0.2%) participants, respectively, received other regimens (predominantly incomplete or NRTI-only records) and are not shown. ^c^Regimen distributions reflect calendar-time shifts in national ART guidelines, notably the programmatic transition to dolutegravir-based first-line therapy, rather than differences attributable to citizenship or migration status alone. ^d^Among migrants, the NVP-based (*p* = 0.363) and Missing (*p* = 0.738) rows show no significant change between eras.

### 3.4 Trends in baseline CD4 cell counts at ART initiation by year and citizenship

**Figure 3:**
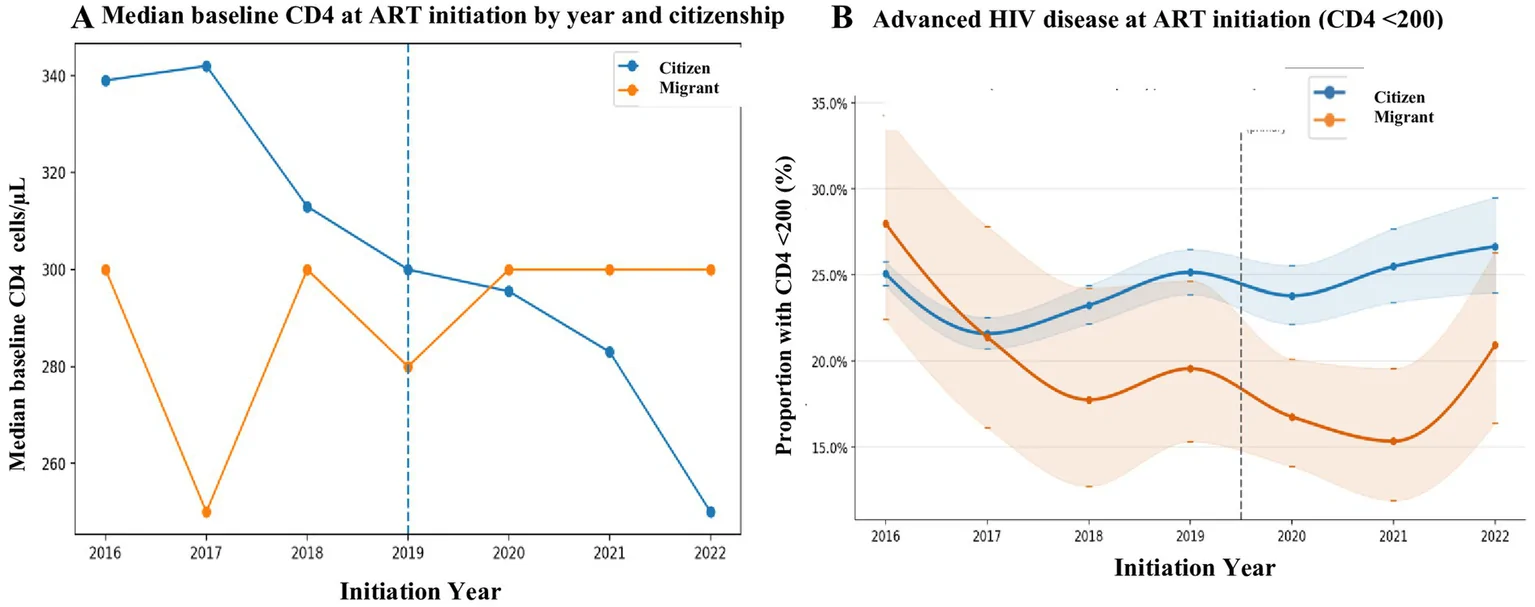
**(A)** Median baseline CD4 cell count at ART initiation stratified by year and citizenship; **(B)** proportion of CD4 cell count <200 cells/μL (%) at ART initiation by citizenship and initiation year.

Median baseline CD4 cell count at ART initiation was relatively high pre-2019 (approximately 339–341 cells/μL), followed by a decline post-2019, where it reached its lowest point of approximately 250 cells/μL. In contrast, migrants displayed inconsistent trends pre-2019, with CD4 cell counts fluctuating between approximately 250 and 300 cells/μL. Notably, migrants had lower median CD4 cell counts than citizens pre-2019. The median CD4 cell count was similar among migrants and citizens in 2020, followed by a decline among citizens through 2022, while for migrants the median CD4 cell count remained consistent (Figure 3A). Among the citizens, the proportion of individuals initiating ART with advanced HIV disease (CD4 < 200 cells/mm^3^) remained stable around 25% for both pre- and post-2019. However, among the migrants, the proportion of individuals initiating ART with advanced HIV disease (CD4 < 200 cells/ μL) was higher than that of citizens at baseline during pre-2019 at approximately 28%, but declined substantially post-2019, reaching approximately 15% before rising again to around 21% (Figure 3B).

### 3.5 Baseline CD4 cell count trends by sex and age group

**Figure 4:**
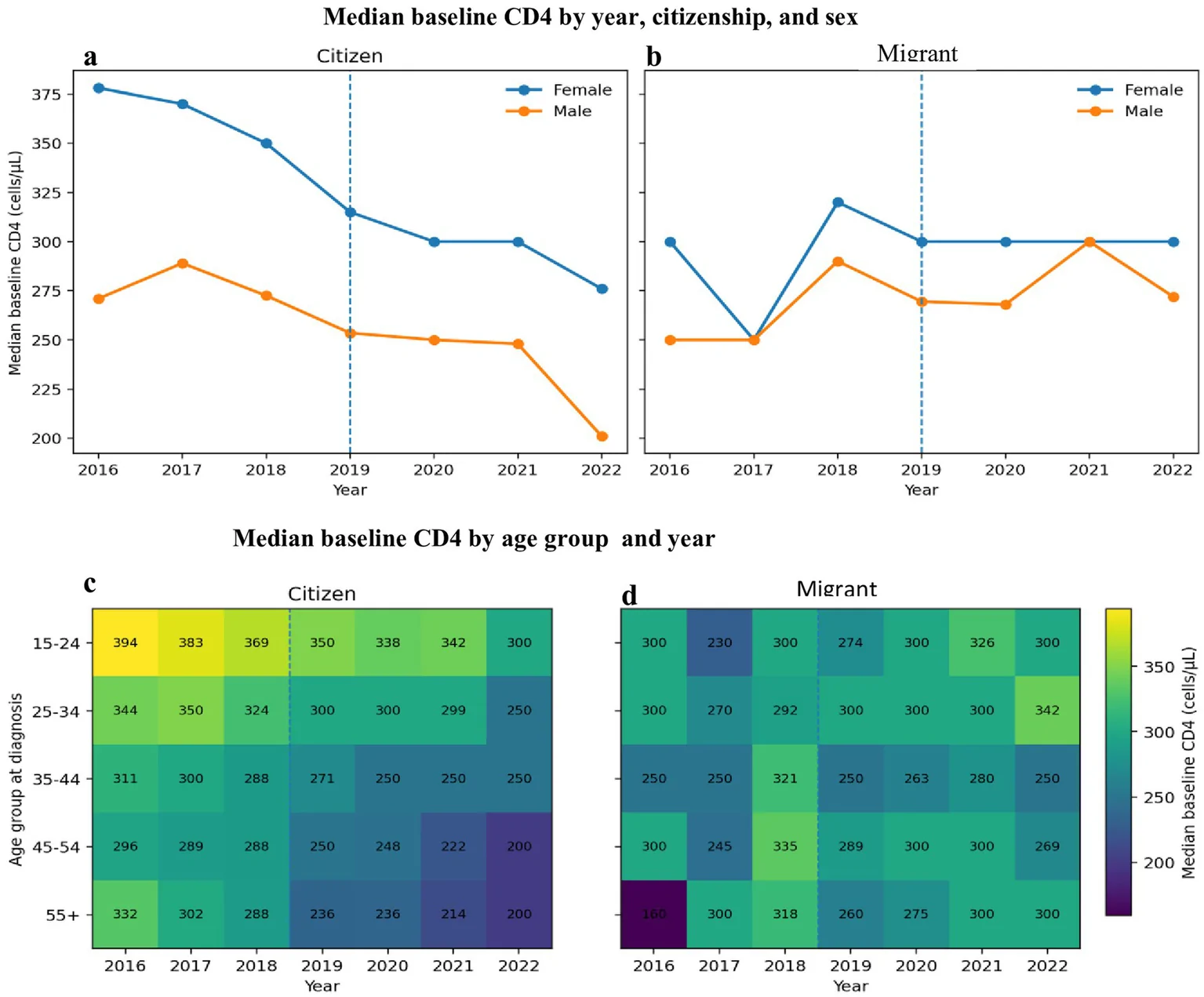
**(a)** Median baseline CD4 cell count by year and sex for citizens; **(b)** median baseline CD4 cell c ount by year and sex for migrants; **(c)** median baseline CD4 cell count by age group and year for citizens; **(d)** median baseline CD4 cell count by age group and year for migrants.

Among citizens, both sexes showed a downward trend over the years (2016–2022). Females consistently initiated ART at higher CD4 cell counts than males, with the median CD4 cell count of females declining from approximately 375 cells/μL in 2016 to 275 cells/μL in 2022, while the male median CD4 cell count declined from approximately 270 cells/μL in 2016 to as low as 200 cells/μL in 2022 (Figure 4a). Among the migrants, the trends were inconsistent for both sex groups pre-2019. Female migrants showed fluctuating CD4 cell counts ranging between 250 and 325 cells/ μL, while male migrants’ CD4 cell counts fluctuated between 250 and 290 cells/μL. Unlike citizens, migrants did not demonstrate a sustained decline in baseline CD4 cell count by sex, though female migrants maintained higher CD4 cell counts compared to males throughout (Figure 4b).

When baseline CD4 cell count is stratified by age group, among citizens, the younger age groups (15–24 and 25–34 years) consistently had the highest CD4 cell counts at ART initiation across all years. However, it has declined over time from 394 cells/μL in 2016 to 300 cells/μL in 2022. Older groups (45–54 and 55+) had lower baseline CD4 cell counts throughout the years. They experienced the steepest declines, with both groups reaching around 200 cells/ μL by 2022, indicating advanced disease at ART initiation (Figure 4c). Among the migrant population, the patterns of CD4 cell counts were more heterogeneous and less consistent when stratified by age. While most age groups fluctuated around 250–300 cells/mm^3^, the 55 + age group in 2016 reported a very low median CD4 of 160 cells/μL, though this may reflect a small sample size. Age group 25–34 showed an increase to 342 cells/μL post-2019. Overall, migrants did not show an age-related decline in baseline CD4 cell counts as observed among citizens (Figure 4d).

### 3.6 Changes in CD4 cell counts using difference-in-differences (DiD) model analysis

When using difference-in-differences models to determine changes in median CD4 cell counts and rates of advanced HIV disease among migrants post-2019, the median change was 54.2 cells/μL (95% CI: 37.7–70.6; *p* < 0.001). After 2019, migrants had 26% lower rates of initiating treatment at advanced HIV stage with CD4 cell counts below 200 cells/μL [0.74 (95% CI: 0.62–0.89; *p* = 0.001)] (Table 3). In supplementary pre-policy diagnostics, annual trends from 2016 to 2019 showed declines in baseline CD4 among both citizens and migrants, with median baseline CD4 falling from 410 to 300 cells/mm^3^ in citizens and from 328 to 300 cells/mm^3^ in migrants (Supplementary Figure S1A). Over the same period, the proportion initiating ART with advanced HIV disease (CD4 < 200 cells/mm^3^) increased from 17.2 to 22.4% among citizens, whereas migrant estimates fluctuated from 20.0% in 2016 to 15.4% in 2019 (Supplementary Figure S1B). In adjusted pre-policy interaction models, there was no strong statistical evidence of differential pre-policy trends between migrants and citizens for continuous baseline CD4 (joint test of migrant × year interactions, *p* = 0.106) or advanced HIV disease at ART initiation (joint test *p* = 0.184). These analyses support, but do not prove, the plausibility of the parallel trends assumption for the baseline CD4-related outcomes.

**Table 3:** Within-migrant pre/post-policy estimates and supportive difference-in-differences interaction estimates for baseline CD4 and advanced HIV disease at ART initiation.

Model/Analysis	Contrast	Estimate	95% CI	*p*-value
Within migrants | baseline CD4, linear regression	Post-2019 vs. pre-2019	1.8 cells/μL	−14.0 to 17.6	0.819
Within migrants | CD4 < 200, modified Poisson	Post-2019 vs. pre-2019	0.76	0.63 to 0.90	0.002
Supportive DiD | baseline CD4, linear regression	Migrant × post-2019	54.2 cells/μL	37.7 to 70.6	<0.001
Supportive DiD | CD4 < 200, modified Poisson	Migrant × post-2019	0.74	0.62 to 0.89	0.001

Models were adjusted for age, sex, baseline CD4 category or continuous baseline CD4 as appropriate, citizenship, policy era, and the citizenship × policy-era interaction. The interaction term represents the difference-in-differences estimate.

### 3.7 Longitudinal trends in the virologic outcomes

**Figure 5:**
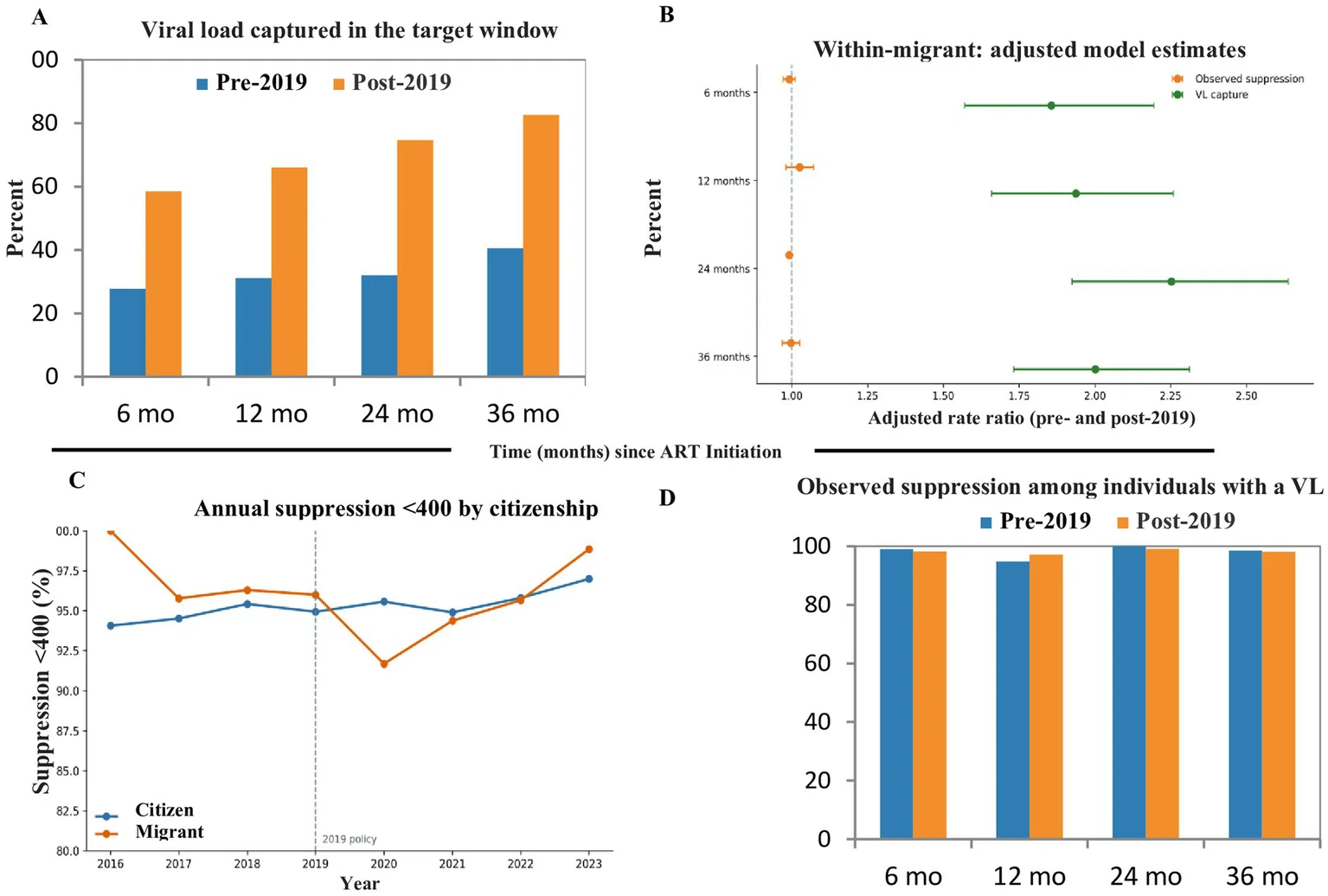
**(A)** Viral load captured over time among migrants pre- and post-policy; **(B)** adjusted relative ratios of VL capture and rates of VL suppression among migrants post-2019 compared to pre-2019 during the follow-up; **(C)** annual VL suppression at < 400 copies/mL by citizenship; **(D)** VL suppression rates over time among migrants.

Among 87,063 included participants, 50,043 (2,086 (4%) migrants and 47,957 (96%) citizens) individuals had VL measurement. Among migrants, VL capture increased from 27.7% before 2019 to 58.5% after 2019 at 6 months, from 31.1 to 66.1% at 12 months, from 32.0 to 74.6% at 24 months, and from 40.5 to 82.6% at 36 months (Figure 5A). When adjusting for being migrant and initiating ART post 2019, adjusted relative ratio of VL capture were increasing overtime post-2019 era (6 months adjusted relative risk [aRR]: 1.86, 95% CI: 1.57–2.20; 12 months: aRR: 1.94, 95% CI: 1.66–2.26; 24 months: aRR: 2.25, 95% CI: 1.92–2.64; 36 months: aRR: 2.00, 95% CI: 1.73–2.31; all *p* < 0.001) (Figure 5B).

The rates of VL suppression at <400 copies/mL were 99.0% before 2019 and 98.2% after 2019 at 6 months; 94.8 and 97.2% at 12 months; 100.0 and 99.1% at 24 months; and 98.5 and 98.1% at 36 months (Figure 5C). The observed suppression among those with a VL remained high in both eras and did not show a consistent post-policy improvement (Figure 5D). When adjusting for citizenship, the adjusted relative ratios showed no association with VL suppression rates changes post 2019 (6 months: aRR: 0.99, 95% CI: 0.97–1.01, *p* = 0.406; 12 months: aRR: 1.03, 95% CI: 0.98–1.07, *p* = 0.266; 24 months: aRR: 0.99, 95% CI: 0.98–1.00, *p* = 0.020; 36 months: aRR: 1.00, 95% CI: 0.97–1.03, *p* = 0.833).

We adjusted for the citizenship and initiating ART post-2019, we reported no association with higher suppression rates at 6, 12, 24, or 36 months (6 months: aRR: 1.00, 95% CI: 0.98–1.02, *p* = 0.705; 12 months: aRR: 1.03, 95% CI: 0.98–1.07, *p* = 0.249; 24 months: aRR: 0.99, 95% CI: 0.98–1.00, *p* = 0.120; 36 months: aRR: 1.00, 95% CI: 0.97–1.03, *p* = 0.849). The annual participant-year graph showed that observed suppression remained high in both citizenship groups throughout follow-up.

### 3.8 Mortality rates

Overall, there were 778 documented deaths among citizens and nine among migrants, leading to crude mortality rates of 0.39 and 0.19 per 100 person-years, respectively. Among migrants, crude mortality was 0.12 per 100 person-years before 2019 and 0.23 per 100 person-years after 2019. However, the follow-up time was substantially shorter in the post-2019 era (median 1.76 vs. 4.72 years). After adjusting for citizenship status and policy era, there was no association between being a migrant and initiating treatment post-2019 and mortality (adjusted hazard ratio [aHR]: 1.99, 95% CI: 0.41–9.70, *p* = 0.393). The post-2020 sensitivity analysis reported a similar association trend (aHR 0.83, 95% CI: 0.22–3.16, *p* = 0.785).

## 4 Discussion

The study evaluated the impact of including migrants in the Botswana national treatment program by expanding the Treat-all policy to cover migrants living in Botswana. Overall, there was a significant increase in the percentage of immigrants initiated on ART post-migrant inclusive treat-all policy from 1.3–4.4% to 19-–0%. The inclusion of migrants in the Treat-all policy was also associated with improved access to HIV treatment and earlier initiation. The observed increase in median CD4 cell counts, reduction in the rates of migrants presenting with advanced HIV disease at ART initiation, and improvement in the VL capture demonstrated the positive outcome associated with the policy inclusion.

Migrants were less likely to initiate treatment with advanced HIV disease after the 2019 policy change, as compared to before 2019. The proportion of migrants presenting with AHD dropped from 22.9% pre-2019–17.7% in the post-2019 period, with a 24% lower risk of advanced disease at ART initiation in the post-policy period, indicating timely and improved access to therapy among migrants. Furthermore, the study revealed that including migrants in the treatment program coincided with a median increase of 54.2 cells/mm^3^ (CI: 37.7 to 70.6; *p*-value <0.001) in CD4 cell counts. This provides strong evidence that the policy changes differentially improved CD4-based outcomes for migrant post-policy change. While the study did not evaluate how the improvements in CD4 cell counts are associated with adverse clinical outcomes, the findings show that widening ART access to migrants was associated with earlier initiation. Several studies have indicated that initiating ART during the acute HIV stage improves the life expectancy of PLWH (21, 22). Two large randomized trials (START and Temprano) reported that PLWH who initiated ART early with high CD4 cell counts have a lower risk of mortality compared to those initiating at an advanced HIV disease stage (23, 24). Therefore, ensuring timely access to ART to all PLWH, regardless of citizenship status, may contribute to improved long-term clinical outcomes and survival probability among migrants, who represent a significant number of PLWH in Botswana.

The study further observed a clear improvement in viral load monitoring among migrants, with increased adjusted relative risks for VL capture ranging from 1.86 to 2.25 across 6–36 months of follow-up. Furthermore, viral suppression among migrants was already high before the policy expansion and remained high (>95%) after the policy expansion without any statistically significant improvement in the observed suppression. This finding shows that the last 95% in the UNAIDS 95-95-95 targets are met even when migrants are included, which is important for sustaining high suppression rates in Botswana. This indicates that prior exclusion from the treatment program revealed the limited routine VL monitoring among a few migrants who could afford treatment services from their own pocket (7, 9, 25).

The mortality rate among migrants was scarce (crude mortality rates of 0.39 per 100 person-years). Among the migrants, crude mortality was 0.12 per 100 person-years before 2019 and 0.23 per 100 person-years after 2019. Migrants would go back home if they are not feeling well, and there could be an underreporting of their mortality in this regard, as they would not be captured when they are outside Botswana. Our study reported no clear evidence of a mortality difference among migrants; therefore, mortality analyses should be interpreted cautiously because only nine documented deaths were observed among migrants. A study done in Yunnan, China, found that mobility was associated with lower mortality among PLWH due to a strong desire to seek treatment and avoid potential HIV/AIDS stigma (26). However, some studies have reported an increased mortality risk among migrants, and this also corresponded to migrants with higher rates of loss to follow-up (7, 8, 27, 28). The factors that are associated with mortality are late presentation at advanced HIV disease stage, HIV-related stigma, and limited access to care (26–28). In our study, the proportions of advanced HIV disease reduced post-2019; however, the rates of mortality were not declining as expected. The elevated crude mortality rates may be attributed to the significantly shorter follow-up period in the post-2019 period compared with the pre-2019 period (median follow-up duration: 1.76 vs. 4.72 years). Several studies have shown that higher rates of mortality are reported in the first year of ART compared to later years on ART (27, 29, 30), showing that a shorter follow-up period may be associated with higher mortality rates, which stabilize with longer follow-ups.

The improved outcomes reported in our study align with modeling studies and recent research on migrant health following inclusion in the Treat-all policy. Marukutira *et.al* 2019, modeled the impact of including migrants in Botswana’s HIV response, reporting scaled-up treatment and care for both citizens and migrants, leading to reduced infections and mortality rates by 44 and 39%, respectively (19). Another study in Botswana compared birth outcomes among migrants and citizens, showing that including ART coverage and extending antenatal care to all pregnant women, regardless of citizenship status, reduces adverse birth outcomes in migrants (8). Previous studies and our study support the hypothesis that providing HIV services to migrants in Botswana could lead to further reductions in HIV incidence and deaths, with scale-up optimized to reach and improve the 95-95-95 fast-track targets by 2030 (4, 17, 31, 32). Despite these improvements, some migrants continue to face challenges in accessing health care adequately, and there are limited effective strategies used to promote health and reach all MLWH (13). This highlights the need for future research to test the effectiveness of interventions for improving testing and treatment uptake, retention, and adherence to ART for all PLWH independent of disease stage (12, 17, 33, 34). Our results highlight the benefit and importance of extending HIV treatment and care services to marginalized populations such as migrants, which was further indicated by the positive impact of the policy shift (35). These findings are important and support migrant inclusive policy as a critical strategy for minimizing structural and policy barriers to care, promoting equity in treatment access, and strengthening the performance of national HIV treatment and care programs.

Several limitations should be acknowledged in this study. The use of real-world data can lead to selection bias when selecting the PLWH who meet study inclusion criteria, leading to some PLWH being excluded from the study. However, PLWH were excluded if their citizenship status, age, CD4 cell count, and/or viral load were missing. Citizenship status was based on the routinely recorded national database citizenship field and categorized as citizen vs. migrant. Misclassification is possible if citizenship was incorrectly recorded or updated incompletely or if individuals had naturalized in the course of treatment. Almost half of the excluded participants had missing baseline CD4 cell count and VL measurement, with low viral load capture among the migrants before the policy change. CD4 cell count was most often undocumented or missing due to the severe shortages of testing reagents nationwide, affecting routine monitoring, and CD4 is no longer the main part of the criteria for ART initiation (36, 37). Missing VL measurements could be due to missed visits, missed tests, or general loss to follow-up. We did not perform a sensitivity analysis to determine the impact of missingness, as PLWH were removed based on the availability of VL measurement or CD4 cell count, and each analysis was based on the population meeting objective inclusion criteria. However, we retained relatively large numbers for the analysis. The mortality rates were lower among migrants, leading to wide confidence intervals for relative ratios, which indicated the impact of small sample size in this analysis. This hindered the ability to detect meaningful differences in mortality rates among migrants and citizens. As an observational analysis of routine program data, the study remains vulnerable to residual confounding from unmeasured factors, including socioeconomic status, adherence, mobility and migration-related vulnerabilities, and differential access to healthcare services. Despite all the limitations, the major strength of this analysis is the use of both cross-sectional and longitudinal approaches across a routinely collected large dataset reflecting real-world service delivery. This created an opportunity to robustly evaluate the impact of the migrant-inclusive national HIV ART program on clinical outcomes among citizens and migrant populations.

## 5 Conclusion

Overall, the findings demonstrated improvements in access to HIV services for migrants post-2019, with a significant impact seen in earlier ART initiation and improved access to viral load monitoring, while observed suppression among those with a VL remained high in both eras. The most immediate policy benefits observed included service access, care engagement, and prevention of late presentation with advanced HIV disease among migrants. Eliminating citizenship-based barriers by providing HIV services to migrants in Botswana significantly improved access to treatment and early initiation. The study warrants continual inclusion of migrants in the Botswana national treatment program with intensified HIV care and monitoring, including routine viral load testing with strong adherence support incorporated. This is a necessity in reducing HIV incidence and adverse clinical outcomes, thereby promoting universal health and supporting epidemic control toward the UNAIDS 95–95–95 targets and ending the HIV epidemic by 2030.

## Data Availability

The data analyzed in this study is subject to the following licenses/restrictions: The dataset contains potentially identifiable participant information. However, available upon specific request with IRB approval. Requests to access these datasets should be directed to sgaseitsiwe@gmail.com.
