# Supplementary material for: The impact of migrant-inclusive policy on Botswana’s universal HIV treatment program: a retrospective analysis of the national HIV treatment program data (2016–2022)
Source: Front Public Health. 2026 Aug 24;14:1909661. doi: 10.3389/fpubh.2026.1909661 (PMC13548394; doi:10.3389/fpubh.2026.1909661)
Supplement: Supplementary file 1 [file Table_1.DOCX]

| Outcome | Interaction term | Estimate | 95% CI | P value |
| --- | --- | --- | --- | --- |
| Continuous baseline CD4 at ART initiation | Migrant × 1 (vs 2016) | 9.8 cells/mm³ | -50.3 to 69.9 | 0.750 |
| Continuous baseline CD4 at ART initiation | Migrant × 1 (vs 2017) | 57.4 cells/mm³ | -2.1 to 116.9 | 0.059 |
| Continuous baseline CD4 at ART initiation | Migrant × 1 (vs 2018) | 49.1 cells/mm³ | -1.7 to 100.0 | 0.058 |
| Continuous baseline CD4 at ART initiation | Joint test of migrant × year interactions (2017–2019) | χ²(3)=6.13 | n/a | 0.106 |
| Advanced HIV disease at ART initiation (CD4 <200 cells/mm³) | Migrant × 1 (vs 2016) | 0.81 RR | 0.44 to 1.49 | 0.496 |
| Advanced HIV disease at ART initiation (CD4 <200 cells/mm³) | Migrant × 1 (vs 2017) | 0.50 RR | 0.25 to 1.02 | 0.057 |
| Advanced HIV disease at ART initiation (CD4 <200 cells/mm³) | Migrant × 1 (vs 2018) | 0.61 RR | 0.34 to 1.08 | 0.090 |
| Advanced HIV disease at ART initiation (CD4 <200 cells/mm³) | Joint test of migrant × year interactions (2017–2019) | χ²(3)=4.84 |  | 0.184 |

# Supplementary Table S1. **Pre-policy interaction tests for baseline CD4 outcomes**

Models were restricted to the pre-policy period (2016-2019) and adjusted for age and sex. For continuous baseline CD4, linear regression with robust standard errors was used. For advanced HIV disease at ART initiation (CD4 <200 cells/mm³), modified Poisson regression with robust standard errors was used. The interaction terms compare migrants with citizens for each pre-policy year relative to 2016. The joint p-values test whether all migrant × year interactions during 2017-2019 are equal to zero.
